# Noninvasive Neuromodulation for Cognitive Dysfunction in Parkinson’s Disease: A Scoping Review

**DOI:** 10.3390/brainsci16101086

**Published:** 2026-10-09

**Authors:** Shelby Chase Loftis, Morgan McClellan, Anna Lise Sheridan, Cynthia Rando, Reginald B. O’Hara

**Affiliations:** 1 Applied Health and Performance, Sophic Synergistics, LLC, 18050 Saturn Ln, Ste 280, Houston, TX 77058, USA; morgan.n.mcclellan@gmail.com (M.M.); rbohara@utep.edu (R.B.O.); 2 College of Science and Mathematics, University of North Georgia, 82 College Circle, Dahlonega, GA 30597, USA; alsher2163@ung.edu; 3 College of Health Sciences, University of Texas at El Paso, 500 W University Ave, El Paso, TX 79968, USA

**Keywords:** Parkinson’s disease, mild cognitive impairment, neuromodulation, vagus nerve stimulation, transcranial direct current stimulation

## Abstract

**Highlights:**

**What are the main findings?**

- Only 4 of 11 studies provided direct PD-MCI evidence, and similar protocols produced divergent cognitive outcomes, with executive function and attention the most frequently assessed domains.
- Some studies pairing neuromodulation with cognitive training reported favorable outcomes, but the pattern was inconsistent across designs and does not establish superiority over standalone stimulation, while no VNS study enrolled confirmed PD-MCI with cognition prespecified as a primary endpoint.

**What are the implications of the main findings?**

- Future trials should enroll confirmed PD-MCI samples and test stimulation with and without cognitive training in adequately powered factorial or multi-arm designs, using sham controls, prespecified domain-sensitive endpoints, and follow-up long enough to test durability.
- Inconsistent reporting of stimulation site, intensity, frequency, and dose prevents pooling, leaving noninvasive VNS an open question rather than a negative result.

**Abstract:**

Background/Objectives: Parkinson’s disease with mild cognitive impairment (PD-MCI) represents a critical early-intervention window before progression to dementia. However, non-pharmacological cognitive treatment options are poorly characterized. This scoping review maps noninvasive neuromodulation interventions evaluated for cognitive outcomes in PD-MCI and in broader PD populations. Methods: Following JBI scoping review methodology and PRISMA-ScR guidelines, PubMed, Scopus and Google Scholar were searched for empirical studies published between May 2006 and September 2026 that applied noninvasive neuromodulation in adults with PD-MCI or broader PD populations and reported cognitive outcomes. Studies were classified as direct PD-MCI evidence (diagnosis confirmed using Movement Disorder Society Task Force criteria) or adjacent PD cognitive evidence. Results: Eleven studies across five modalities were included: tDCS (*n* = 6), iTBS (*n* = 2), TPS (*n* = 1), tcVNS (*n* = 1), and taVNS (*n* = 1). Only four studies (two tDCS and two iTBS) enrolled participants with confirmed PD-MCI; the remaining seven enrolled broader PD populations. Favorable findings came mainly from small samples, and protocols, outcome measures, and populations were heterogeneous. Conclusions: Evidence specific to PD-MCI is limited and mixed. Several studies pairing stimulation with cognitive training reported favorable outcomes, but this pattern was inconsistent and does not establish superiority over stimulation alone. No VNS trial enrolled participants with confirmed PD-MCI or used cognition as a primary endpoint.

## 1. Introduction

Parkinson’s disease (PD) is a progressive neurodegenerative disorder affecting 1–2% of the population over 65 years of age and is the second most common neurodegenerative disease after Alzheimer’s disease (AD) [1,2]. Mild cognitive impairment may occur in Parkinson’s disease and is associated with an increased risk of progression to Parkinson’s disease dementia (PDD), although not all individuals with Parkinson’s Disease-Mild Cognitive Impairment (PD-MCI) develop dementia [3]. In the past, cognitive treatment during the PD-MCI stage was undervalued because of its relatively minor impact on quality of life compared with the moderate and severe stages of PD and PDD [3]. However, recent studies have identified the PD-MCI stage as the earliest clinical indication of PDD [3], demonstrating the need for non-invasive methods to prevent cognitive decline in patients with PD-MCI [4].

The Movement Disorder Society (MDS) Task Force defines PD-MCI at two diagnostic levels [5]. Level I is an abbreviated assessment based on a validated global cognitive screening scale or a limited neuropsychological battery. Level II is a comprehensive assessment with at least two tests in each of the five cognitive domains: (1) attention and working memory, (2) executive function, (3) language, (4) memory, and (5) visuospatial function. A Level II diagnosis requires impairment on at least two tests, either within one domain or across two domains [5]. In this review, studies were classified as direct PD-MCI evidence when all participants met these criteria at Level I or Level II (Section 2.3). Using the MDS criteria, PD-MCI can be identified earlier in “at-risk” patients, for clinician monitoring and assessment of emerging interventions [3].

PDD is a severe neurodegenerative condition characterized by progressive alpha-synuclein aggregation forming Lewy bodies and Lewy neurites across cortical and subcortical regions [6]. The impact of dementia on a movement disorder such as Parkinson’s can place an immense physical, financial, and psychological burden on the patient and caregiver, further affecting their quality of life. An estimated 75% of people with PD who survive more than 10 years develop PDD, and patients with PD-MCI are 6.5 times more likely to develop dementia within five years after PD diagnosis [6].

Due to the wide range of treatment drugs available for various presentations of PD, combinations of motor and cognitive medications are often prescribed together [6]. Patients with PD may require prescription medications for multiple motor symptoms, nonmotor symptoms, and comorbid conditions. Polypharmacy, defined as the concurrent use of five or more medications, has been associated with drug-to-drug interactions, poor treatment adherence, hospitalization, poor quality of life, frailty, cognitive impairment, and mortality [7,8]. While these do not establish that polypharmacy directly induces progression from PD-MCI to PDD, there are concerns suggesting the necessity for future investigation of nonpharmacological interventions to supplement pharmacological treatments.

Recent studies suggest non-invasive neuromodulation as a potential PD-MCI treatment intervention that may provide patients with a nonpharmacological adjunct treatment modality to mitigate cognitive dysfunction and possibly reduce contraindications with polypharmacy [9,10,11,12,13,14,15]. New advancements in neural stimulation techniques are targeting the loss of dopaminergic neuron function in PD to modulate neural activity within networks implicated in cognitive dysfunction in at-risk patients [6]. These treatments seek to reduce neurodegeneration effects, such as memory loss, by modulating neural activity in specific cortical regions to improve cognition during the PD-MCI stage [6,16]. With limited and poorly quantified empirical evidence, there is still no clear understanding of how neuromodulation directly affects cognitive function in patients with PD-MCI.

Various non-invasive treatment techniques have been proposed; however, research findings have not been synthesized to provide a comprehensive understanding of their effects on cognitive outcomes. Hence, the purpose of this scoping review is to map non-invasive neuromodulation studies reporting cognitive outcomes relevant to PD-MCI, with particular attention to stimulation parameters, cognitive domains assessed, and evidence gaps related to vagus nerve stimulation (VNS), including transcutaneous cervical (tcVNS) and transcutaneous auricular (taVNS) approaches.

The objective of this review is to map the range of available evidence on noninvasive interventions for cognitive dysfunction in PD with emphasis on PD-MCI, by characterizing intervention modalities, stimulation parameters, cognitive outcomes, and current evidence gaps.

The primary question of this scoping review is (1) What noninvasive neuromodulation interventions have been evaluated for cognitive outcomes in adults with confirmed PD-MCI? A sub-question is (2) What adjacent evidence exists from broader PD populations, including studies of tcVNS and taVNS?

## 2. Materials and Methods

This scoping review was conducted in accordance with the Joanna Briggs Institute (JBI) methodology for scoping reviews [17] and is reported in accordance with the Preferred Reporting Items for Systematic Reviews and Meta-Analyses Extension for Scoping Reviews (PRISMA-ScR) [18]. The completed PRISMA-ScR checklist is provided as Supplementary Table S1. An a priori protocol was developed and approved by all authors prior to execution of the search strategy. The protocol was not registered but is available from the corresponding author upon request.

### 2.1. Inclusion Criteria

Eligibility criteria were organized according to the JBI Types of Participants, Concept, Context, and Types of Evidence Sources and were supplemented by prespecified exclusion criteria [17,19].

Population: Eligible studies included adults diagnosed with PD-MCI. Due to the limited empirical evidence specific to PD-MCI, studies involving broader PD populations that also reported cognitive outcomes relevant to PD-MCI were eligible. Each included study was classified as direct PD-MCI evidence or adjacent PD cognitive evidence using the criteria described in Section 2.3.

Concept: Studies were required to examine noninvasive neuromodulation interventions delivered through external electrical, magnetic, or acoustic stimulation devices. Eligible modalities included transcranial direct current stimulation (tDCS), intermittent theta-burst stimulation (iTBS), repetitive transcranial magnetic stimulation (rTMS), transcranial pulse stimulation (TPS), and transcutaneous vagus nerve stimulation (VNS). Studies also had to report cognitive outcomes pertaining to PD-MCI and describe stimulation parameters and intervention protocols.

Context: Studies conducted in clinical, outpatient, rehabilitation, or research settings were eligible for inclusion. No geographic restrictions were applied. Only peer-reviewed articles published in the English language between May 2006 and September 2026 were considered. The start date was predetermined to capture approximately the previous 20 years of research, and the end date corresponds to the final search on 21 September 2026.

Types of Evidence Sources (Inclusion Criteria): Studies were required to report cognitive outcomes relevant to PD-MCI, including global cognition or one or more cognitive domains (attention and working memory, executive function, language, memory, or visuospatial function). Studies reporting stimulation parameters, protocol characteristics, and associated non-cognitive outcomes were also eligible when cognitive outcomes were included. Eligible study designs included quantitative experimental and quasi-experimental studies, including randomized controlled trials (RCTs), crossover studies, parallel-group studies, open-label pilot studies, and observational cohort studies.

Exclusion Criteria: Studies were excluded if they were not peer-reviewed, were not published in English, did not examine a noninvasive neuromodulation intervention, did not report cognitive outcomes pertinent to PD-MCI or PD cognitive dysfunction, or were published outside the predetermined search period (May 2006–September 2026).

### 2.2. Search Strategy and Screening

The authors (SCL, ALS) performed a systematic literature search according to the Population, Concept, Context (PCC) framework recommended by JBI for all scoping reviews [19]. An initial limited database search was conducted across PubMed and Google Scholar for studies related to the topic, objectives, and review questions of this review. For the second and final database search, we used text words identified from the titles and abstracts of relevant articles along with database-specific index terms used to describe those articles in PubMed, Google Scholar, and Scopus. The final searches were executed on 21 September 2026 for all databases. Limits applied in each database were publication date (May 2006 to September 2026) and English language. Scopus and Google Scholar are restricted by publication year only, so the range 2006 to 2026 was applied in those databases and the May 2006 start date was applied during screening. Records from all three databases were exported together for deduplication; per-database record counts were not retained at the time of the search, and the combined total was 12,290 records. The database-specific search strategies, including controlled vocabulary, truncation, and limits, are reported in Supplementary Text S1. These were reconstructed from the documented search terms and limits because the executed strings were not archived.

The Population portion combined terms associated with PD and mild cognitive impairment, including (“Parkinson’s Disease” OR “Parkinsonian disorders”) AND (“mild cognitive impairment” OR “PD-MCI”). The Concept component included terms associated with noninvasive neuromodulation, including (“noninvasive neuromodulation” OR “brain stimulation” OR “transcranial direct current stimulation” OR “transcranial magnetic stimulation” OR “theta-burst stimulation” OR “intermittent theta-burst stimulation” OR “transcranial pulse stimulation” OR “vagus nerve stimulation” OR “transcutaneous vagus nerve stimulation”). Terms that were synonymous within each concept were then combined using the Boolean operator OR, and the Population and Concept components were combined using AND.

Database-specific controlled vocabulary was used where available, along with free text terms and truncation strategies appropriate for each of the database searches. Since this scoping review also sought to locate adjacent PD populations reporting cognitive outcomes pertinent to PD-MCI, title and abstract screening was used to identify broader PD sources that met the eligibility criteria even when PD-MCI terminology was not precisely reported. Lastly, the reference lists of all included studies and relevant systematic reviews and meta-analyses were manually screened and forward citation tracking was conducted to locate additional eligible sources not retrieved through the electronic database searches.

### 2.3. Source Selection, Data Extraction, and Synthesis

Records were imported into Microsoft Excel (version 2609; Microsoft Corporation, Redmond, WA, USA) and Mendeley Reference Manager (version 2.149.0; Mendeley Ltd., London, UK) to aid with deduplication, screening, and data extraction. Duplicates were identified automatically in Mendeley, then verified manually in Excel by matching title, author, year, and DOI. Three reviewers (SCL, MM, ALS) independently screened titles and abstracts against the inclusion criteria and then independently assessed the full texts of potentially eligible articles. Disagreements relevant to study eligibility were resolved through discussion until a consensus was reached.

A data extraction form was constructed by SCL. Each reviewer pilot-tested the form on a minimum of three full-text articles included in the review, after which the form was revised prior to full data extraction. Data were extracted by SCL and independently verified for accuracy and completeness by MM. The final data extracted from each included study were (1) author and year, (2) study design, (3) sample size, (4) evidence classification, (5) criteria used to define participant cognitive status, (6) intervention modality and any adjunct training, (7) stimulation target, (8) stimulation parameters, (9) protocol characteristics, (10) cognitive domains assessed, and (11) key cognitive findings.

Studies were classified as direct PD-MCI evidence when all participants met MDS Task Force criteria for PD-MCI [5] at Level I or Level II, as reported by the study authors. Studies enrolling broader idiopathic or nondemented PD populations that reported cognitive outcomes without formal PD-MCI confirmation, including studies that characterized cognition using other screening thresholds or classification schemes, were classified as adjacent PD cognitive evidence. Since iTBS is a patterned form of rTMS, studies delivering iTBS were classified as iTBS regardless of the terminology used by the original authors.

The extracted data were summarized using descriptive and narrative synthesis methods consistent with the scoping review objective of mapping the available evidence. Study characteristics were tabulated, and findings were organized by evidence classification, intervention modality, cognitive domain, stimulation target, protocol characteristics, and relevance to the review questions. Lastly, studies were categorized to describe patterns across included sources, including modality type, population characteristics, cognitive outcomes examined, and direction of reported outcomes. Consistent with JBI guidance for scoping reviews [19], formal critical appraisal and risk-of-bias assessment were not performed. Accordingly, the synthesis describes the direction of reported findings and does not weight studies by methodological quality or estimate the comparative efficacy of modalities. Adverse events and tolerability were not systematically extracted, and this review therefore does not draw conclusions regarding safety.

## 3. Results

Figure 1 depicts the scoping review flowchart for selecting sources. Articles were screened for eligibility based on the established inclusion criteria. Of the 154 full-text articles examined for eligibility, 143 were excluded, and 11 primary empirical studies were included in the evidence synthesis. Nine studies were identified through database searches and two through citation tracking of a recent meta-analysis of transcutaneous VNS (tVNS) in PD [20].

### 3.1. Characteristics of Included Studies

The 11 studies included were published between 2006 and 2026 and were conducted across multiple countries. All were primary empirical studies: 10 were randomized controlled trials [9,10,11,12,13,15,21,22,23,24], including three crossover trials [9,10,22], and one was an open-label pilot study [14]. Sample sizes ranged from 9 [10] to 49 participants [15]. The studies covered five modalities: tDCS (*n* = 6) [9,10,11,12,21,22], iTBS (*n* = 2) [13,23], TPS (*n* = 1) [14], tcVNS (*n* = 1) [24], and taVNS (*n* = 1) [15]. Conventional rTMS was an eligible modality (Section 2.1), but no included study delivered it. Both studies of patterned magnetic stimulation delivered iTBS and are classified as iTBS (Section 2.3). Four studies incorporated structured cognitive training. In two, all arms received cognitive training and active stimulation was compared with sham [12,21]; in the other two, separate arms allowed combined treatment to be compared with stimulation alone [11,13]. Study characteristics are summarized in Table 1, grouped by evidence classification, and stimulation protocols are summarized in Table 2, grouped by modality.

Using the criteria described in Section 2.3, four studies were classified as direct PD-MCI evidence (Biundo et al. [21], Lawrence et al. [11], Lang et al. [23], and Cheng et al. [13]) and seven as adjacent PD cognitive evidence (Boggio et al. [9], Ishikuro et al. [10], Manenti et al. [12], Lau et al. [22], Gianlorenco et al. [14], Sigurdsson et al. [24], and Wang et al. [15]).

### 3.2. Mapping of Evidence

The 11 studies included examined five noninvasive neuromodulation modalities (Table 2) [9,10,11,12,13,14,15,21,22,23,24]. Among the studies included, four enrolled participants with confirmed PD-MCI and seven assessed broader PD populations reporting cognitive outcomes relevant to PD-MCI.

The left dorsolateral prefrontal cortex (L-DLPFC) was the most frequently targeted cortical region, used in seven of the nine transcranial stimulation studies [9,11,12,13,21,22,23]. VNS interventions targeted either the cervical vagus nerve [24] or the auricular branch of the vagus nerve [15]. Stimulation parameters, treatment schedules, and follow-up periods varied across studies and are summarized in Table 2.

Executive function and attention were each assessed in 9 of 11 studies, memory or working memory in 9, global cognition in 6, and language and visuospatial function in 4 each (Table 1). Considerable heterogeneity was observed across participant populations, intervention protocols, outcome measures, and study designs, which limited direct comparison across modalities.

### 3.3. Direct PD-MCI Evidence

Four studies, two of tDCS and two of iTBS, enrolled participants with confirmed PD-MCI [11,13,21,23], and their findings were evenly divided. Lawrence et al. reported improvements across several domains in arms receiving cognitive training, tDCS, or both, relative to control [11], and Cheng et al. reported improvements in global cognition and delayed memory when iTBS was combined with VR cognitive training, relative to both sham and iTBS alone [13]. In contrast, Biundo et al. found no immediate benefit of active tDCS added to cognitive training, with sham outperforming active stimulation on a written coding task [21], and Lang et al. found no between-group differences after correction for multiple comparisons [23]. No TPS or VNS study enrolled participants with confirmed PD-MCI.

### 3.4. Adjacent PD Cognitive Evidence

Seven studies enrolled broader PD populations [9,10,12,14,15,22,24]. Favorable between-group findings were reported by Boggio et al. (three-back accuracy after a single 2 mA session) [9], Ishikuro et al. (TMT-A completion time) [10], and Manenti et al. (phonemic fluency, with both arms receiving cognitive training) [12]. Gianlorenco et al. reported within-group improvement in an uncontrolled open-label design [14]. Lau et al. [22], Sigurdsson et al. [24], and Wang et al. [15] reported no between-group cognitive effects.

## 4. Discussion

The objective of this review was to map and characterize the available evidence on noninvasive neuromodulation interventions for cognitive dysfunction in PD, with a central focus on PD-MCI. Specifically, this review aimed to identify which neuromodulation modalities have been studied, the stimulation protocols and cognitive outcomes that have been reported, and the current evidence gaps that exist relevant to PD-MCI.

Three overarching findings emerged. First, the cognitive effects of noninvasive neuromodulation were heterogeneous, with similar stimulation protocols often producing varying cognitive outcomes. Second, the L-DLPFC emerged as a predominant target across transcranial neuromodulation studies, reflecting its proposed role in executive dysfunction but without direct comparative evidence supporting its superiority over alternate targets [9,11,12,13,21,22,23]. Third, although two VNS studies met the inclusion criteria, neither enrolled participants with confirmed PD-MCI, highlighting an important evidence gap for this target population. Collectively, these findings indicate that noninvasive neuromodulation is a promising but preliminary area of research for cognitive dysfunction in PD-MCI and that more well-designed clinical trials are necessary before definitive conclusions can be made regarding its effectiveness and safety. Formal risk-of-bias assessment was not performed and only four studies enrolled participants with confirmed PD-MCI, meaning that the patterns described below should be read as descriptive features of a small, heterogeneous evidence base rather than as evidence of comparative efficacy. The evidence classification of each study is stated throughout to distinguish direct PD-MCI evidence from adjacent PD evidence.

### 4.1. Transcranial Direct Current Stimulation (tDCS)

Of all modalities, tDCS was the most extensively investigated, representing six of the eleven included studies [9,10,11,12,21,22]. Two of these enrolled participants with confirmed PD-MCI [11,21], and four enrolled broader PD populations [9,10,12,22]. tDCS modulates cortical excitability by applying weak direct current through scalp electrodes, with anodal stimulation generally increasing and cathodal stimulation generally decreasing neuronal membrane excitability [25]. The L-DLPFC was selected as the anodal target in five of the six studies because of its established role in working memory, executive function, and top-down cognitive control, domains commonly impaired in PD-MCI [9,11,12,21,22].

Four of the six tDCS studies reported a between-group improvement in at least one cognitive outcome [9,10,11,12], and two reported null or unfavorable effects [21,22]. However, this majority-positive signal must be interpreted cautiously, as three of the four positive studies provided adjacent rather than direct PD-MCI evidence, several had small samples, and most reported effects on one or a few outcomes among many assessed (Section 4.6.3). A notable comparison is provided by Boggio et al. [9] and Lau et al. [22], both adjacent evidence, who administered a single session of anodal L-DLPFC tDCS at 2 mA for 20 min using crossover designs but reported different cognitive outcomes. Boggio et al. observed significant improvement in a three-back working memory task, while Lau et al. found no significant effects on visual working memory or go/no-go performance. Differences in the cognitive outcome measures, participant cognitive status, and concurrent treatment interventions may have contributed to these divergent results. Lau et al. also noted that their working memory task was administered at the start of stimulation, as opposed to Boggio et al., who assessed performance during the final minutes of stimulation [22]. However, because multiple variables differed simultaneously, the specific source of the discrepancy cannot be determined.

A complementary contrast is provided by Biundo et al. [21] and Lawrence et al. [11], the two tDCS studies providing direct PD-MCI evidence, both of which used 4-week tDCS protocols targeting the L-DLPFC at 2 mA. The studies differed in session frequency, four sessions per week in Biundo et al. versus one per week in Lawrence et al., and in design. In Biundo et al., both arms received cognitive training and active tDCS was compared to sham [21], while Lawrence et al. used a multi-arm design that included stimulation-only, training-only, and combined arms alongside a control group [11]. Neither publication specified whether the treatment consisted of spaced or massed stimulation, warranting further investigation. The results also differed, with Biundo et al. finding no significant immediate benefit of active tDCS, with sham outperforming active stimulation on a written coding task at 4 weeks, and only non-significant trends favoring active tDCS on memory measures at 16-week follow-up [21]. Lawrence et al. reported significant improvements across multiple cognitive domains, with more outcomes improving in arms that combined cognitive training with tDCS [11]. These contrasting results are compatible with, but do not demonstrate, a contribution of concurrent cognitive engagement. Since the two studies differed in session frequency, design, outcome measures, and sample characteristics, the source of the difference cannot be isolated.

Manenti et al. [12], an adjacent evidence study, compared active with sham tDCS, each delivered during cognitive training in 22 participants with PD [12]. Both groups improved on global cognition, semantic fluency, action naming, and several executive measures, and only phonemic fluency improved significantly more with active tDCS. With both arms having received cognitive training and the design not including stimulation-only or training-only arms, this study estimates the added effect of tDCS on cognitive training and cannot determine whether the combination is superior to either intervention alone. Among the tDCS studies, only Lawrence et al. included the separate arms needed for that comparison [11]. Ishikuro et al. [10], also adjacent evidence, extended the target site literature by applying anodal tDCS over the frontal polar area (FPA) rather than the L-DLPFC, observing shorter TMT-A completion time relative to sham at immediate follow-up, although the pairwise comparison reached only *p* = 0.05 [10]. While preliminary, this finding suggests that prefrontal targets outside the DLPFC may warrant further investigation in PD-related cognitive dysfunction. However, the small sample size (*n* = 9) and pilot nature of the study mean that this finding requires replication in larger study cohorts. Together, these results indicate that the current evidence for tDCS in PD-MCI is limited and heterogeneous, with direct PD-MCI evidence restricted to two studies with divergent results, and that larger, methodologically standardized trials are needed before conclusions can be drawn regarding clinical efficacy.

### 4.2. Intermittent Theta-Burst Stimulation (iTBS)

Two studies, both providing direct PD-MCI evidence, investigated iTBS targeting the L-DLPFC and reported contrasting cognitive outcomes [13,23]. Lang et al. [23] conducted a double-blind, sham-controlled RCT in 41 participants with PD-MCI meeting MDS Task Force Level II criteria. No significant cognitive improvement was observed on any primary or secondary outcome across five cognitive domains at one-month follow-up. A non-significant trend toward improved executive function was observed but did not survive correction for multiple comparisons [23]. These null findings are clinically informative because all participants had confirmed PD-MCI. Nevertheless, the findings are limited to the specific protocol evaluated and therefore should not be interpreted as evidence that iTBS is ineffective for PD-MCI [23].

Cheng et al. [13] randomized 40 participants with PD-MCI to iTBS combined with VR cognitive training (*n* = 13), iTBS alone (*n* = 11), or sham (*n* = 16) [13]. Although the original authors described the intervention as rTMS, stimulation was delivered as iTBS over the L-DLPFC at 90% RMT across 10 consecutive sessions and is classified here as iTBS (Section 2.3). The combined iTBS and VR group improved significantly relative to sham on RBANS total score, delayed memory, MoCA total score, and MoCA visuospatial/executive score; iTBS alone exceeded sham only on MoCA language [13]. As with the combined tDCS studies, this finding raises the possibility that concurrent cognitive training may enhance neuromodulation effects, but the arms were small, and the contribution of VR training independent of stimulation could not be estimated because the design did not include a VR-only arm [13].

The discrepancy between Lang et al. [23] and Cheng et al. [13] may reflect differences in the number of sessions (6 vs. 10), stimulation intensity (80% aMT vs. 90% RMT), and the presence of adjunct VR cognitive training. Because several protocol variables differed simultaneously, their individual contributions cannot be isolated. Thus, future controlled trials should examine stimulation parameters and adjunct cognitive training independently [13,23].

### 4.3. Transcranial Pulse Stimulation (TPS)

TPS was represented by a single open-label pilot study, which precludes definitive conclusions but does provide preliminary clinical observations and exploratory neurophysiological findings that warrant discussion [14]. TPS delivers focused, ultra-short acoustic shockwave pulses to targeted brain regions. Its proposed actions include mechanotransductive effects and modulation of neural activity. However, these mechanisms remain incompletely established [14].

In this study, 14 participants aged 50 to 78 years with clinically established or probable PD spanning Hoehn and Yahr stages 2 to 4 received 12 TPS sessions over four weeks. The multicomponent protocol included bilateral M1 stimulation, individualized symptom-targeted cortical regions, standardized whole-cortex stimulation, and peripheral plantar stimulation, with serial assessments during treatment and at one-month follow-up [14]. Participants were not enrolled based on a confirmed PD-MCI diagnosis. This study therefore provides adjacent PD cognitive evidence and has limited direct applicability to the target population of this review. Overall cognitive performance, a secondary outcome measured using the Scales for Outcomes in PD-Cognition (SCOPA-COG), a PD-specific neuropsychological instrument that examines multiple cognitive domains, improved 25% from baseline, with the largest gain observed by session three [14]. Concurrent changes in resting EEG oscillatory activity were also observed. However, the relationship between neurophysiological and clinical findings remains uncertain [14].

These findings are hypothesis-generating but must be interpreted within the constraints of an open-label design, the absence of a sham-control group, small sample size (*n* = 14), an individualized multicomponent protocol, repeated cognitive testing, a heterogeneous cohort, and a limited one-month follow-up [14]. Expectancy effects, practice effects, regression to the mean, and the contribution of individual protocol components cannot be excluded. Sham-controlled randomized trials enrolling participants with confirmed PD-MCI are needed before conclusions relevant to the efficacy of TPS for PD-MCI-related cognitive dysfunction can be drawn. Nonetheless, TPS is a technically distinct stimulation modality from tDCS and iTBS and requires further examination in controlled trials using standardized PD-MCI diagnostic criteria and prespecified cognitive outcomes.

### 4.4. Vagus Nerve Stimulation (VNS)

VNS represents a mechanistically distinct neuromodulation approach and constitutes one of the principal evidence gaps identified in this review. The vagus nerve is the tenth cranial nerve and a major component of the parasympathetic nervous system. Approximately 80% of its fibers are afferent and transmit sensory information to the nucleus tractus solitarii (NTS), which has downstream projections to neuromodulatory centers including the locus coeruleus (LC), dorsal raphe nucleus, and basal forebrain [26,27,28]. Through these projections, VNS is posited to influence distributed cortical networks through modulation of noradrenergic, serotonergic, and cholinergic signaling, although the precise mechanisms are incompletely understood [27,28]. This pathway may be relevant to PD-MCI because degeneration of the nucleus basalis of Meynert and associated cholinergic dysfunction have been implicated in deficits involving attention, executive function, and memory [29].

Noninvasive transcutaneous VNS is delivered through two commonly studied approaches: (1) tcVNS, applied over the cervical vagus nerve, and (2) taVNS, applied to auricular sites innervated by the auricular branch of the vagus nerve [15,24]. taVNS typically targets auricular regions such as the tragus or cymba conchae and is intended to activate afferent vagal pathways [30,31]. These pathways project to brainstem nuclei and may influence distributed cortical regions involved in cognitive and affective processing [32,33], although these effects have not been well elucidated in PD-MCI populations. Afferent vagal pathways have been examined as one possible mechanism through which taVNS may influence autonomic and cognitive processes [33].

Beyond PD, taVNS has been studied across several neurological and psychiatric disorders, providing preliminary evidence that vagal stimulation may influence cognitive, affective, and autonomic outcomes [27,28,29,30,31,32,33,34]. Stimulation intensity and other dosing parameters may influence neuromodulatory effects [35], although the optimal stimulation sites, intensities, and dosing protocols have not been established [35,36].

#### 4.4.1. Findings from Included VNS Studies

Two VNS studies met the inclusion criteria. Both provide adjacent PD evidence because neither study enrolled participants with confirmed PD-MCI, limiting direct applicability to the target population of this scoping review [15,24].

Sigurdsson et al. [24] conducted a single-center, double-blind, sham-controlled proof-of-concept RCT of domiciliary tcVNS in 33 participants with PD. Participants self-administered two 120 s stimulations twice daily at 25 Hz for 12 weeks [24]. The primary objective was feasibility, acceptability, adherence, and safety, with cognition as a secondary, exploratory outcome [24]. Computerized measures of attention and Cambridge Neuropsychological Test Automated Battery (CANTAB) measures of executive function and visual memory showed no statistically significant treatment effects relative to sham in the primary analysis.

Wang et al. [15] conducted a single-blind, sham-controlled RCT of home-based bilateral taVNS in 49 participants with PD (Hoehn and Yahr stages 1 to 3). Stimulation was delivered to the cymba conchae at 5 Hz for 30 min twice daily for three weeks [15]. The taVNS group showed significant improvements compared with sham in autonomic symptoms, sleep disturbance, and PD-specific quality of life, evaluated using the Scales for Outcomes in PD-Autonomic Dysfunction (SCOPA-AUT), PD Sleep Scale-2 (PDSS-2), and 39-item PD Questionnaire (PDQ-39). However, no significant between-group difference was observed in global cognition assessed using the Montreal Cognitive Assessment (MoCA), which was not the primary outcome.

Neither study reported significant cognitive benefit, and treatment duration cannot be isolated as the explanatory factor because the trials also differed in stimulation route, frequency, dose, participant characteristics, and cognitive outcome measures [15,24]. The tcVNS study primarily used domain-specific computerized assessments, differing from the taVNS study, which used a brief global cognitive screening measure. Although brief global measures may be less sensitive than comprehensive neuropsychological test batteries for detecting small domain-specific cognitive changes [4,37], measurement sensitivity alone cannot fully explain the null findings across both studies. As such, the current evidence does not establish that VNS is ineffective for cognitive dysfunction in PD-MCI, as neither study showed a significant cognitive benefit under the protocols tested, and neither enrolled participants with confirmed PD-MCI or assessed cognition as the primary endpoint [15,24]. Future investigations should enroll participants with confirmed PD-MCI, prespecify cognition as a primary outcome variable, use adequately powered samples and sensitive domain-specific neuropsychological measures, and systematically examine stimulation parameters.

#### 4.4.2. VNS Translational Potential

Current research indicates that noninvasive VNS for cognitive dysfunction in PD-MCI is at an early translational stage, with the available empirical evidence derived mainly from broader PD populations rather than from individuals with confirmed PD-MCI [15,24]. The theoretical rationale for noninvasive VNS is based on its proposed engagement of afferent vagal projections to the NTS in the medulla, with downstream modulation of neuromodulatory systems linked with cognition [26,28]. Preliminary evidence suggests that stimulation parameters, including intensity, could influence physiological and neurophysiological responses, but the optimal stimulation sites and dosing parameters have not been adequately established [35].

Although neither Wang et al. nor Sigurdsson et al. reported significant cognitive improvement relative to sham [15,24], Wang et al. did report changes in regional brain activity and white matter integrity following taVNS despite the absence of measurable cognitive improvement [15]. These findings suggest biological engagement of neural pathways but do not establish that these neurophysiological changes translate into clinically significant cognitive improvement. Additionally, preliminary evidence from other PD samples suggests that transcutaneous VNS may improve selected cognitive processes, such as response time and processing speed, but these outcomes cannot be generalized to patients with confirmed PD-MCI [38]. Collectively, these findings support the need for systematic dose-optimization studies and adequately powered sham-controlled clinical trials examining whether VNS-induced physiological changes correspond to clinically relevant improvements in cognition [15,24,35,36,38], informed by dosing experience from taVNS research in other conditions such as chronic pain, depression, and autonomic regulation [39,40,41].

### 4.5. Cross-Study Patterns

Three cross-cutting patterns were observed across modalities. Given the absence of formal quality appraisal and the small number of studies, these are descriptive observations rather than conclusions about comparative efficacy. First, four studies incorporated structured cognitive training, and their results were mixed. In the two studies whose designs allowed combined treatment to be compared with stimulation alone, both with small arms, combined treatment showed more favorable outcomes on some measures [11,13]. In the two studies in which all arms received cognitive training, adding active stimulation produced a between-group benefit on a single outcome [12] or no immediate benefit [21]. Since these studies varied in protocol design, the available evidence does not establish that concurrent cognitive training enhances the effects of neuromodulation. These observations support formal testing of stimulation-augmented cognitive rehabilitation in PD-MCI rather than its adoption [11,12,13,21].

Second, single-session crossover designs cannot determine whether cumulative or durable effects emerge following repeated neuromodulation sessions [9,22]. The heterogeneous results observed in these studies, both of which provided adjacent evidence, together with their reliance on immediate post-stimulation assessments, suggest that such designs may be better suited for examining acute proof-of-concept effects than durable clinical efficacy.

Third, the convergence on L-DLPFC as a stimulation target across seven of nine transcranial neuromodulation studies reflects a theoretically grounded but empirically narrow approach. Executive dysfunction is a prominent feature of PD-MCI, providing a rationale for L-DLPFC targeting [42]. However, PD-MCI may also involve impairments in memory, visuospatial processing, and attention that are supported by distributed neural networks (e.g., posterior parietal, medial temporal, and striatal) extending beyond the prefrontal cortex [42]. The individualized, multi-site targeting approach employed by Gianlorenco et al. [14] represents a conceptually distinct strategy that may address this network-level complexity. Nonetheless, its clinical effects remain uncertain because the approach has been examined in only a small sample open-label pilot study with no sham-control group [14].

### 4.6. Research Gaps

#### 4.6.1. Population Gaps

A fundamental challenge in synthesizing this literature is the inconsistency in the definitions and confirmation of cognitive impairment across studies. Of the 11 included studies, four enrolled populations formally confirmed as PD-MCI using MDS Task Force criteria [11,13,21,23]. The remaining seven studies enrolled broader idiopathic PD or non-demented PD populations diagnosed using established PD criteria and, in some studies, characterized by Hoehn and Yahr stage, but without formal confirmation of PD-MCI [9,10,12,14,15,22,24]. Consequently, these broader PD samples included participants with varying baseline cognitive profiles, introducing clinical heterogeneity and uncertainty in cognitive status classification. Some studies applied cognitive screening thresholds. For instance, Lau et al. [22] required a Mini-Mental State Examination (MMSE) score of ≥ 26 [22] but did not positively confirm MCI, and Manenti et al. (2018) characterized cognitive status using PD-CRS cut-off scores rather than MDS criteria [12].

This lack of diagnostic specificity in participant selection means that several studies may have enrolled populations spanning cognitively unimpaired PD and varying degrees of cognitive impairment without formal PD-MCI classification. Such heterogeneity may have obscured differences in treatment response according to baseline cognitive status.

Therefore, it is difficult to determine whether reported effects apply specifically to PD-MCI or reflect broader cognitive changes across mixed PD populations. Future clinical trials of neuromodulation should prioritize rigorous PD-MCI confirmation using standardized MDS diagnostic criteria to improve population specificity and comparability across studies [5].

Baseline cognitive status may also interact with stimulation effects. Participants with little or no cognitive impairment have limited room to improve on standard neuropsychological measures, so ceiling effects could attenuate between-group differences in broader PD samples. Descriptively, the included studies do not show a clear gradient by baseline status. Among the four direct PD-MCI studies, two reported a favorable between-group effect on at least one outcome [11,13] and two reported none [21,23]. Among the seven adjacent PD studies, three reported a favorable between-group effect on at least one outcome [9,10,12], three reported none [15,22,24], and one reported within-group change only [14]. Only Lau et al. examined response as a function of baseline performance and found no difference between low and high baseline performers, although a sample of 10 participants provided little power for this comparison [22]. Because baseline cognitive scores, outcome measures, and protocols differed across studies and individual participant data were unavailable, response trajectories by baseline impairment could not be compared formally. Ceiling effects remain a plausible but untested explanation for some null findings in broader PD samples. Future trials could address this by stratifying randomization by baseline cognitive status, prespecifying baseline cognition as a moderator, and selecting outcome measures without ceiling constraints in mildly impaired samples.

#### 4.6.2. Methodological Gaps

Sample sizes were generally modest, ranging from 9 to 49 participants, with six of the 11 studies enrolling fewer than 30 participants [9,10,12,14,21,22]. Only Lang et al. (2020) reported an a priori power calculation, estimating that 32 participants per group were required, but did not reach the planned sample due to recruitment issues [23]. These sample limitations may have reduced statistical power and the ability to detect modest effects in cognition. Short-term follow-up in several studies also limited the ability to determine whether the reported cognitive benefits were durable or primarily acute post-intervention effects [9,11,14,22,23].

Blinding integrity was not consistently assessed or reported across sham-controlled studies. Differences in sensation between active and sham stimulation could allow participants to infer treatment allocation and introduce expectancy bias, but this possibility cannot be confirmed without formal assessment [9,10,11,12,15,21,22,23,24]. There was also considerable methodological heterogeneity across study designs. For instance, designs included crossover trials, parallel-group RCTs, and an open-label pilot study [9,10,11,12,13,14,15,21,22,23,24]. These designs differ in their susceptibility to carryover, expectancy, selection, and other sources of bias, complicating cross-study comparisons and interpretation of cognitive outcomes. Lastly, the reliance on single-center designs in several studies may limit generalizability across clinical settings and the broader PD population.

#### 4.6.3. Statistical Validity of Reported Findings

The favorable findings in this literature should be interpreted in light of their statistical limitations. Small samples reduce the power to detect true effects, and they also lower the probability that a statistically significant result reflects a true effect and inflate the magnitude of effects that do reach significance [43]. Several favorable findings came from the smallest samples, including a crossover study of 9 participants with a pairwise comparison at *p* = 0.05 [10], a single-session crossover study of 18 participants [9], an uncontrolled open-label study of 14 participants [14], and multi-arm trials with approximately 7 to 16 participants per arm [11,13]. Most studies assessed numerous cognitive outcomes, often at multiple time points, and correction for multiple comparisons was not consistently reported. Under these conditions, the probability that at least one outcome reaches significance by chance is substantial. Lang et al. applied such correction, and an uncorrected executive function trend did not survive it [23], illustrating how conclusions can depend on this step. In addition, the large effect sizes reported in some studies, such as d = 1.16 to 1.27 for within-group change in the open-label TPS study [14], are likely to be overestimates in samples of this size. No included study appears to have been adequately powered for a prespecified primary cognitive endpoint in participants with confirmed PD-MCI. For these reasons, the favorable findings summarized in this review are best regarded as hypothesis-generating, and the null results from sham-controlled trials of comparable or larger size [15,23,24] warrant equal consideration in guiding future research.

#### 4.6.4. Outcome Measurement Gaps

Neuropsychological outcome instruments were heterogeneous across studies, and no single battery was used consistently across the evidence base. Brief global cognitive measures were used in some studies, and the outcome approaches differed within the VNS literature, with one study using the MoCA and the other using domain-specific computerized assessments [15,24]. Although brief global screening tools such as the MoCA and MMSE are useful for evaluating cognitive status, they may have limited sensitivity to subtle, domain-specific changes in mild cognitive impairment when used alone [4,37].

Comprehensive outcome assessment spanning all five MDS-defined cognitive domains [5] (attention/working memory, executive function, memory, visuospatial, and language) was uncommon, limiting the ability to determine whether interventions differentially affected specific cognitive functions relevant to PD-MCI. This heterogeneity complicates cross-study comparisons and future quantitative synthesis and limits interpretation of null findings, which may reflect an absence of intervention effect, limited instrument sensitivity, or other methodological factors. Future trials could adopt a Level II battery with at least two tests per MDS domain, using the validated tests recommended by the MDS Task Force [5], and report domain composite z-scores alongside individual test results. A shared battery of this kind would allow direct cross-study comparison and support quantitative synthesis as the evidence base grows.

#### 4.6.5. Stimulation Protocol Gaps

Even within modalities, stimulation parameters varied substantially. For example, tDCS studies used current intensities of 1.0 to 2.0 mA and session durations of 15 to 25 min, while treatment schedules ranged from single-session protocols to once weekly and up to five-times-weekly sessions [9,10,11,12,21,22]. Since stimulation intensity, duration, and treatment schedule influence physiological responses to tDCS [25], variation in these parameters may have contributed to the inconsistent cognitive outcomes observed across included studies [9,10,11,12,21,22]. However, the independent contribution of each parameter cannot be isolated from differences in participant characteristics, outcome measures, and adjunct interventions.

Both iTBS studies used 50 Hz bursts delivered at 5 Hz and 600 pulses, although differing in intensity setting method (e.g., 80% aMT vs. 90% RMT), session count (6 vs. 10), and intervention conditions, with only Cheng et al. (2022) incorporating adjunct VR cognitive training [13,23]. These differences make it difficult to determine whether the contrasting findings were associated with protocol characteristics, adjunct cognitive training, participant factors, or other study-level differences. TPS provided limited opportunity for parameter optimization or protocol comparison because evidence was restricted to a single open-label pilot study without sham control or alternative dosing conditions [14].

The two VNS studies differed in stimulation frequency, session duration, total treatment duration, and delivery route (tcVNS vs. taVNS) [15,24]. Given the small number of studies and the simultaneous differences between protocols, the null cognitive findings cannot be attributed to targeting, dosing, or any single stimulation parameter. Differences in stimulation targets among the included modalities further limited direct cross-study comparisons, although such differences are expected among interventions designed to target distinct neural pathways [9,10,11,12,13,14,15,21,22,23,24]. Future empirical investigations should prioritize standardized, modality-specific protocol reporting, and systematic dose-finding studies addressing stimulation intensity, treatment frequency, target selection, and duration, which would improve comparability across studies and support more rigorous examination of cognitive outcomes.

### 4.7. Strengths and Limitations

The structured search across PubMed, Scopus, and Google Scholar, supplemented by citation tracking, provided broad coverage of the available literature. Use of JBI guidance and PRISMA-ScR reporting recommendations further enhanced methodological transparency [17,18,19]. However, methodological quality and risk of bias were not formally evaluated, consistent with JBI guidance for scoping reviews [19]. Therefore, the findings were not weighted according to study quality, and the descriptive contrasts between favorable and null findings in this review should not be read as evidence of comparative efficacy. The inclusion of both cognitive and non-motor data, combined with detailed extraction of stimulation protocols, resulted in an evidence map that may inform future trial design [9,10,11,12,13,14,15,21,22,23,24].

Limitations include restriction to peer-reviewed English-language publications, which may have excluded studies published in other languages and unpublished or gray literature. The executed search strings and per-database record counts were not retained, and the strategies reported in the Supplementary Text S1 were reconstructed retrospectively from the documented search terms and limits, which limits the complete reproducibility of the search. Several studies enrolled broader PD populations without formal confirmation of PD-MCI, limiting direct applicability of the findings to the target population. Only four included studies enrolled participants with PD-MCI formally confirmed using MDS Task Force criteria [11,13,21,23], limiting confidence in conclusions specific to PD-MCI. There was also considerable heterogeneity in cognitive outcome measures, participant characteristics, study designs, and stimulation parameters [9,10,11,12,13,14,15,21,22,23,24]. The evidence included in this review was derived from only 11 studies, many with small sample sizes and relatively short or varying follow-up periods, limiting the generalizability of the findings [9,10,11,12,13,14,15,21,22,23,24]. Adverse events and tolerability were not systematically extracted, so no conclusions regarding safety can be drawn. Medication state at the time of cognitive assessment (ON or OFF dopaminergic medication) and concurrent anticholinergic use were inconsistently reported by the primary studies, so their influence on cognitive outcomes could not be evaluated. This is an unmeasured source of variability across the included trials because cognitive performance in PD can fluctuate with medication state. With this scoping review being restricted to peer-reviewed articles, publication bias cannot be excluded. This review was designed to map and characterize the available evidence rather than quantitatively examine comparative effectiveness or safety. Hence, the findings presented in this review should be interpreted as an evidence map identifying areas for future empirical research rather than definitive evidence that supports clinical implementation.

### 4.8. Future Research Directions

The findings of this scoping review highlight several directions for future research in noninvasive neuromodulation for PD-MCI. Across modalities, future trials would benefit from prioritizing populations with PD-MCI confirmed using MDS Task Force criteria [5], adopting standardized, domain-specific neuropsychological batteries aligned with the five MDS-defined cognitive domains (Section 4.6.4), standardizing and reporting medication state at each assessment, prespecifying and powering a primary cognitive endpoint, systematically reporting adverse events and tolerability, and including follow-up beyond immediate post-intervention assessment, extending over several months where feasible, to better assess durability of cognitive outcomes. Within each modality, standardized protocol reporting and systematic parameter comparison should address the variability that currently limits cross-study comparison and synthesis [9,10,11,12,13,14,15,21,22,23,24]. This is particularly relevant for tDCS, for which session frequency and the co-administration of cognitive training remain potential sources of heterogeneity rather than established moderators [9,10,11,12,21,22]. Some studies combining neuromodulation with cognitive training reported favorable cognitive outcomes with tDCS and iTBS [11,12,13], although findings were not consistent across all combined protocols [21]. These results support adequately powered factorial or multi-arm trial designs for estimating the independent and interactive contributions of stimulation and cognitive training, rather than examining them only as combined intervention packages [11,12,13,21].

For VNS, addressing the evidence gaps remains an important research priority. Until sham-controlled RCTs specifically enroll participants with confirmed PD-MCI and prespecify cognition as a primary outcome, the population-specific evidence gap identified by this scoping review will remain [15,24]. Early-phase studies examining safety, tolerability, feasibility, and dose optimization in participants with confirmed PD-MCI are needed before larger efficacy trials are undertaken. Such studies should monitor verbal fluency, given a reduction reported in one small left-ear taVNS trial summarized in a recent meta-analysis, although the duration, reproducibility, and clinical significance of this finding are uncertain [20].

Home-based tVNS delivery may reduce the burden of repeated clinical visits and has demonstrated preliminary feasibility and acceptability in broader PD populations; however, its accessibility and suitability for individuals with PD-MCI still need direct evaluation [15,24]. Longer-duration trials incorporating neuroimaging or other target engagement biomarkers may help assess whether VNS-associated physiological changes correspond to clinically meaningful cognitive outcomes [15]. Future VNS studies should also consider systematic comparisons of cervical and auricular delivery, stimulation site and laterality, and individualized targeting approaches [15,24,35].

More broadly, these directions are consistent with a previous therapeutic approach, in which stimulation targets and parameters are individualized and matched to the mechanisms underlying cognitive dysfunctions in each patient. Lastly, progress in neuromodulation research for PD-MCI will likely be strengthened by standardized diagnostic criteria, rigorous trial methodology, systematically assessed stimulation parameters, and clinically meaningful cognitive outcome measures [5].

## 5. Conclusions

This scoping review mapped noninvasive neuromodulation interventions for cognitive dysfunction in PD, with emphasis on PD-MCI, identifying 11 studies across five modalities (tDCS, iTBS, TPS, tcVNS, and taVNS) published between 2006 and 2026 [9,10,11,12,13,14,15,21,22,23,24]. Only four studies, two of tDCS and two of iTBS, enrolled participants with PD-MCI formally confirmed using MDS Task Force criteria [11,13,21,23], and their findings were evenly divided between favorable and null results. The remaining seven studies enrolled broader PD populations, and their findings cannot be assumed to apply to PD-MCI. Across both groups, results were heterogeneous and derived largely from small samples, and no formal risk-of-bias assessment was performed. Some studies combining neuromodulation with cognitive training reported favorable cognitive outcomes [11,13], but this pattern was inconsistent [12,21] and does not establish superiority over stimulation alone. No included VNS study enrolled participants with confirmed PD-MCI or examined cognition as a primary outcome; consequently, the cognitive effects of VNS in PD-MCI remain undetermined. Future studies should confirm PD-MCI using standardized diagnostic criteria, use adequately powered sham-controlled designs with prespecified primary cognitive endpoints, standardize and report medication state, include sensitive domain-specific neuropsychological outcomes, incorporate sufficient follow-up to assess durability, and systematically report stimulation parameters and adverse events. Overall, noninvasive neuromodulation remains an investigational approach for cognitive dysfunction in PD-MCI and requires more rigorous trials before conclusions can be made regarding clinical efficacy or implementation.

## Figures and Tables

**Figure 1:**
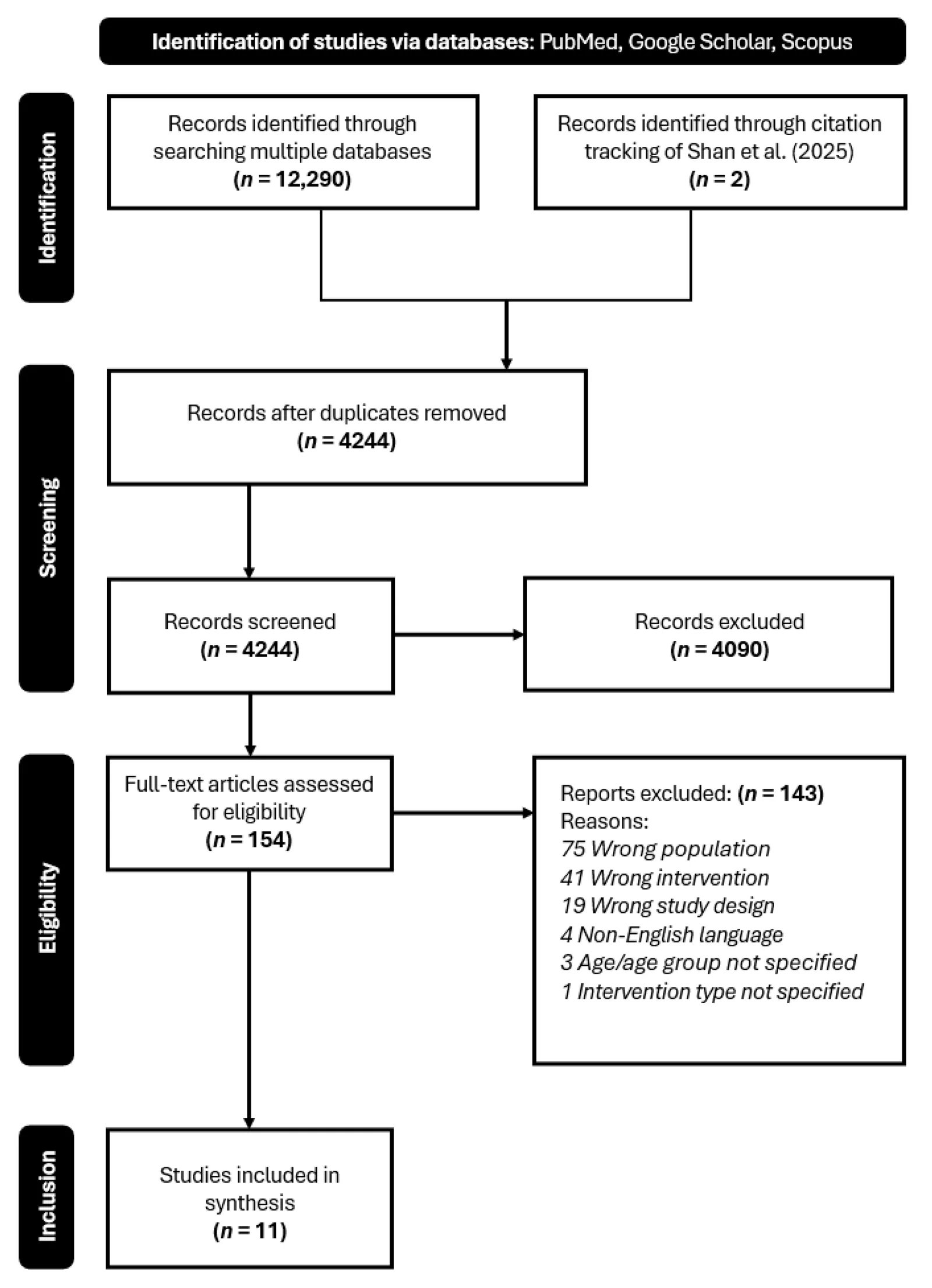
Scoping review flowchart [18]. Records identified through citation tracking of Shan et al. [20] were screened together with database records. Duplicates (*n* = 8048) were removed as described in Section 2.3.

**Table 1:** Characteristics of included studies, grouped by evidence classification.

Study [Ref.]	Design	*n*	Modality	Evidence Classification	Cognitive Status criteria	Cognitive Domains Assessed	Key Cognitive Findings
Direct PD-MCI evidence (*n* = 4)							
Biundo et al. (2015) [21]	RCT, sham-controlled, double-blind	24	tDCS + CT (CT in both arms)	Direct PD-MCI	MDS Task Force Criteria Level II	Executive function, attention, working memory, memory, visuospatial	Sham outperformed real tDCS on written coding at 4 weeks (*p* = 0.001, d = 1.52). Non-significant trends favored real tDCS at 16 weeks on story learning (*p* = 0.077) and the immediate memory index (*p* = 0.075). No other between-group differences.
Lawrence et al. (2018) [11]	RCT, parallel group, multi-arm	42	tDCS, CT, and tDCS + CT arms	Direct PD-MCI	MDS Task Force Level II	Global cognition, executive function, attention, working memory, memory, language, visuospatial	Standard CT plus tDCS improved SOC (*p* < 0.001), Stroop (*p* < 0.001), and Similarities (*p* = 0.01). Tailored CT plus tDCS improved SOC (*p* < 0.001), letter-number sequencing (*p* = 0.01), Paragraph Recall (*p* < 0.001), and Similarities (*p* < 0.001). tDCS alone improved Stroop (*p* = 0.02) and Paragraph Recall (*p* < 0.001). No arm improved global cognition or visuospatial performance.
Lang et al. (2020) [23]	RCT, sham-controlled, double-blind	41	iTBS	Direct PD-MCI	MDS Task Force Level II	Global cognition, executive function, attention, memory, language, visuospatial	No between-group differences in any domain after correction for multiple comparisons. An uncorrected trend favored real iTBS on executive function at 1 month (*p* = 0.042). Language and visuospatial gains were driven by the sham arm.
Cheng et al. (2022) [13]	RCT, sham-controlled	40	iTBS + VR CT; iTBS alone	Direct PD-MCI	MDS Task Force Level II	Global cognition, executive function, attention, working memory, memory, language, visuospatial	After treatment, iTBS + VR exceeded sham on RBANS total (*p* < 0.001), delayed memory (*p* = 0.001), MoCA total (*p* = 0.012), and MoCA visuospatial/executive (*p* = 0.008), and exceeded iTBS alone on RBANS total (*p* < 0.001), delayed memory (*p* < 0.001), and MoCA visuospatial/executive (*p* = 0.046). iTBS alone exceeded sham on MoCA language only (*p* = 0.024). At 3 months, iTBS + VR remained superior to sham on RBANS total (*p* = 0.014) and delayed memory (*p* = 0.002), and to iTBS alone on delayed memory (*p* = 0.028).
Adjacent PD cognitive evidence (*n* = 7)							
Boggio et al. (2006) [9]	RCT, sham-controlled, crossover	18	tDCS	Adjacent PD	Idiopathic PD; no formal MCI classification	Attention, working memory	Anodal L-DLPFC tDCS at 2 mA improved three-back accuracy relative to both sham and M1 stimulation and reduced errors relative to sham (all *p* < 0.05). The 1 mA dose did not affect accuracy (*p* = 0.61). Neither dose affected reaction time.
Ishikuro et al. (2018) [10]	RCT, sham-controlled, crossover	9	tDCS	Adjacent PD	PD; no formal MCI classification	Executive function	Anodal tDCS shortened TMT-A completion time relative to sham (omnibus H = 7.801, *p* = 0.02; pairwise *p* = 0.05). Cathodal tDCS did not differ from sham.
Manenti et al. (2018) [12]	RCT, sham-controlled, double-blind, parallel group	22	tDCS + CT (CT in both arms)	Adjacent PD	UK PD Society Brain Bank criteria; cognitive status classified using PD-CRS cut-off scores	Global cognition, executive function, attention, working memory, language	Phonemic fluency improved more with active than sham tDCS (F(1,20) = 4.43, *p* = 0.048). PD-CRS, semantic fluency, action naming, Stroop, and response flexibility improved in both arms with no between-group difference. No memory effects.
Lau et al. (2019) [22]	RCT, sham-controlled, crossover	10	tDCS	Adjacent PD	Nondemented PD; MMSE ≥ 26	Attention, working memory	No effect of anodal tDCS relative to sham on visual working memory (*p* = 0.80), go/no-go reaction time (*p* = 0.87), hit rate (*p* = 0.78), or false-alarm rate (*p* = 0.51). Effects did not differ between low and high baseline performers.
Gianlorenco et al. (2026) [14]	Open-label, single-arm pilot	14	TPS	Adjacent PD	Clinically established or probable PD; H&Y 2 to 4	Global cognition, executive function, attention, working memory	Within-group change only; no comparator arm. SCOPA-COG improved 7.28 points by session 12 (*p* < 0.001, d = 1.16) and 7.43 points at follow-up (d = 1.27).
Sigurdsson et al. (2025) [24]	RCT, sham-controlled, double-blind, parallel group	33 (16/17)	tcVNS	Adjacent PD	PD; PDD or significant cognitive impairment excluded	Executive function, attention, working memory	No between-group effects in the primary analysis on attention (PoA *p* = 0.40, FA *p* = 0.19, PS *p* = 0.73, CoA *p* = 0.96), executive function (OTS-MLFC *p* = 0.13, OTS-PSFC *p* = 0.76), or visual memory (PAL-TEA *p* = 0.42). Per-protocol analysis favored sham on OTS-MLFC (*p* = 0.047), with a time-by-group interaction favoring active tcVNS at follow-up (*p* = 0.016).
Wang et al. (2026) [15]	RCT, sham-controlled, single-blind	49	taVNS	Adjacent PD	PD, H&Y 1 to 3; no formal MCI classification	Global cognition, executive function	No between-group difference in MoCA (*p* = 0.42). Within-group change was non-significant in both arms (taVNS *p* = 0.069, sham *p* = 0.090).

Abbreviations: CT, cognitive training; H&Y, Hoehn and Yahr stage; iTBS, intermittent theta-burst stimulation; L-DLPFC, left dorsolateral prefrontal cortex; M1, primary motor cortex; MMSE, Mini-Mental State Examination; MoCA, Montreal Cognitive Assessment; OTS-MLFC, One Touch Stockings of Cambridge, mean latency to first choice; OTS-PSFC, One Touch Stockings of Cambridge, problems solved on first choice; PAL-TEA, Paired Associative Learning-Total Errors Adjusted; PD-CRS, Parkinson’s Disease Cognitive Rating Scale; PoA, power of attention; FA, fluctuating attention; PS, processing speed; CoA, continuity of attention; RBANS, Repeatable Battery for the Assessment of Neuropsychological Status; RCT, randomized controlled trial; SCOPA-COG, Scales for Outcomes in Parkinson’s disease-Cognition; SOC, Stockings of Cambridge; taVNS, transcutaneous auricular vagus nerve stimulation; tcVNS, transcutaneous cervical vagus nerve stimulation; tDCS, transcranial direct current stimulation; TMT-A, Trail Making Test-Part A; TPS, transcranial pulse stimulation; VR, virtual reality.

**Table 2:** Stimulation protocols across all included studies, grouped by modality.

Study [Ref.]	Target	Intensity	Amplitude (mA)	Frequency (Hz)	Pulse Width	Session Time (m)	Rest Period (m)	Session Frequency (Doses)
tDCS (*n* = 6)								
Biundo et al. (2015) [21]	L-DLPFC	–	2	–	–	20	NR	4×/week for 4 weeks
Boggio et al. (2006) [9]	L-DLPFC	–	1–2	–	–	20	NR	Single session
Ishikuro et al. (2018) [10]	FPA	–	1	–	–	15	NR	5×/week for 1 week per condition
Lau et al. (2019) [22]	L-DLPFC	–	2	–	–	20	NR	Single session per condition
Lawrence et al. (2018) [11]	L-DLPFC	–	2	–	–	20	NR	1×/week for 4 weeks
Manenti et al. (2018) [12]	L-DLPFC	–	2	–	–	25	NR	5×/week for 2 weeks
iTBS (*n* = 2)								
Lang et al. (2020) [23]	L-DLPFC	80% aMT	–	50	NR	NR	NR	6×/week for 1 week
Cheng et al. (2022) [13]	L-DLPFC	90% RMT	–	50	NR	NR	NR	10 consecutive sessions (5×/week for 2 weeks)
TPS (*n* = 1)								
Gianlorenco et al. (2026) [14]	Bilateral M1 + symptom-targeted ROIs	0.25 mJ/mm^2^	–	4	NR	50	NR	3×/week for 4 weeks
tcVNS (*n* = 1)								
Sigurdsson et al. (2025) [24]	Cervical vagus nerve	Individually adjusted	–	25	NR	2 per stimulation	2 to 5	28×/week (2 doses 2×/day) for 12 weeks
taVNS (*n* = 1)								
Wang et al. (2026) [15]	Cymba conchae (bilateral)	Individually adjusted	1.5 to 10	5	500 μs	30	60	2×/day for 3 weeks

Abbreviations: aMT, active motor threshold; FPA, frontal polar area; –, not applicable; NR, not reported; RMT, resting motor threshold; ROI, region of interest. Other abbreviations as in Table 1.

## Data Availability

No new data were created or analyzed in this study. Data sharing is not applicable to this article.

## Supplementary Materials

The following supporting information can be downloaded at: https://www.mdpi.com/article/10.3390/brainsci16101086/s1, Table S1: PRISMA-ScR Checklist; Text S1: Database-specific search strategies.

## Abbreviations

The following abbreviations are used in this manuscript:

AD	Alzheimer’s Disease
aMT	Active motor threshold
CANTAB	Cambridge Neuropsychological Test Automated Battery
CoA	Continuity of attention
CT	Cognitive training
FA	Fluctuating attention
FPA	Frontal polar area
H&Y	Hoehn and Yahr stage
iTBS	Intermittent theta-burst stimulation
JBI	Joanna Briggs Institute
LC	Locus coeruleus
L-DLPFC	Left dorsolateral prefrontal cortex
M1	Primary motor cortex
MDS	Movement Disorder Society
MMSE	Mini-Mental State Examination
MoCA	Montreal Cognitive Assessment
NTS	Nucleus tractus solitarii
NR	Not reported
OTS-MLFC	One Touch Stockings of Cambridge, mean latency to first choice
OTS-PSFC	One Touch Stockings of Cambridge, problems solved on first choice
PAL-TEA	Paired Associative Learning-Total Errors Adjusted
PD	Parkinson’s disease
PD-CRS	Parkinson’s Disease Cognitive Rating Scale
PDD	Parkinson’s disease with dementia
PD-MCI	Parkinson’s disease with mild cognitive impairment
PDQ-39	Parkinson’s Disease Questionnaire-39
PDSS-2	Parkinson’s Disease Sleep Scale-2
PoA	Power of attention
PRISMA-ScR	Preferred Reporting Items for Systematic Reviews and Meta-Analyses extension for Scoping Reviews
PS	Processing speed
RBANS	Repeatable Battery for the Assessment of Neuropsychological Status
RCT	Randomized controlled trial
RMT	Resting motor threshold
ROI	Region of interest
rTMS	Repetitive transcranial magnetic stimulation
SCOPA-AUT	Scales for Outcomes in Parkinson’s disease-Autonomic Dysfunction
SCOPA-COG	Scales for Outcomes in Parkinson’s disease-Cognition
SOC	Stockings of Cambridge
taVNS	Transcutaneous auricular vagus nerve stimulation
tcVNS	Transcutaneous cervical vagus nerve stimulation
tDCS	Transcranial direct current stimulation
TPS	Transcranial pulse stimulation
tVNS	Transcutaneous vagus nerve stimulation
VNS	Vagus nerve stimulation
VR	Virtual reality
