# Supplementary material for: Noninvasive Neuromodulation for Cognitive Dysfunction in Parkinson’s Disease: A Scoping Review
Source: Brain Sci. 2026 Oct 9;16(10):1086. doi: 10.3390/brainsci16101086 (PMC13702581; doi:10.3390/brainsci16101086)
Supplement: Supplementary file 1 [file brainsci-16-01086-s001.zip › supplementary-Text S1.pdf]

Supplementary Text S1.
Database-Specific Search Strategies

This supplement reports the search strategies for each database searched in the scoping review "Noninvasive Neuromodulation for Cognitive Dysfunction in Parkinson's Disease: A Scoping Review." Searches followed the Population, Concept, Context framework. Population terms (Parkinson's disease and cognitive impairment) and Concept terms (noninvasive neuromodulation modalities) were each combined with OR, and the two components were combined with AND.

The exact search strings as executed in each database were not archived at the time of the search. The strategies below were reconstructed from the documented search terms, Boolean structure, and limits reported in Section 2.2 of the manuscript and are provided to allow the search to be reproduced. Per-database record counts and the number of Google Scholar results screened were likewise not retained; the combined total retrieved across the three databases was 12,290 records.

S1.1. Search summary

Database	Date searched	Limits applied	Records retrieved
PubMed (MEDLINE)	September 21, 2026	Publication date 2006/05/01 to 2026/09/21; English	Not retained*
Scopus	September 21, 2026	Publication years 2006 to 2026; English	Not retained*
Google Scholar	September 21, 2026	Custom range 2006 to 2026; sorted by relevance	Not retained*
Total database records	September 21, 2026		12,290
Citation tracking	Not recorded	Citation tracking of Shan et al. (2025)	2

**Per-database record counts were not retained at the time of the search. The combined total retrieved across PubMed, Scopus, and Google Scholar was 12,290 records, as reported in Figure 1. Scopus and Google Scholar restrict by publication year only. The year range 2006 to 2026 was applied in those databases, and records published before May 2006 were excluded during screening under the eligibility criteria (Section 2.1).*

S1.2. PubMed

#1 "Parkinson Disease"[Mesh] OR "Parkinsonian Disorders"[Mesh] OR parkinson*[tiab]

#2 "Cognitive Dysfunction"[Mesh] OR "mild cognitive impairment"[tiab] OR "PD-MCI"[tiab] OR "cognitive impairment"[tiab]

#3 "Transcranial Direct Current Stimulation"[Mesh] OR "Transcranial Magnetic Stimulation"[Mesh] OR "Vagus Nerve Stimulation"[Mesh] OR "Transcutaneous Electric Nerve Stimulation"[Mesh] OR "noninvasive neuromodulation"[tiab] OR "non-invasive neuromodulation"[tiab] OR "brain stimulation"[tiab] OR "transcranial direct current stimulation"[tiab] OR tDCS[tiab] OR "transcranial magnetic stimulation"[tiab] OR rTMS[tiab] OR "theta-burst stimulation"[tiab] OR "theta burst stimulation"[tiab] OR iTBS[tiab] OR "transcranial pulse stimulation"[tiab] OR "vagus nerve stimulation"[tiab] OR "transcutaneous vagus nerve stimulation"[tiab] OR taVNS[tiab] OR tcVNS[tiab] OR tVNS[tiab]

#4 #1 AND #2 AND #3

#5 #4 AND ("2006/05/01"[Date - Publication] : "2026/09/21"[Date - Publication]) AND English[Language]

S1.3. Scopus

TITLE-ABS-KEY ((parkinson* OR "parkinsonian disorder*") AND ("mild cognitive impairment" OR "PD-MCI" OR "cognitive impairment" OR "cognitive dysfunction") AND ("noninvasive neuromodulation" OR "non-invasive neuromodulation" OR "brain stimulation" OR "transcranial direct current stimulation" OR tdcs OR "transcranial magnetic stimulation" OR rtms OR "theta-burst stimulation" OR "theta burst stimulation" OR itbs OR "transcranial pulse stimulation" OR "vagus nerve stimulation" OR "transcutaneous vagus nerve stimulation" OR tavns OR tcvns OR tvns)) AND PUBYEAR > 2005 AND PUBYEAR < 2027 AND (LIMIT-TO (LANGUAGE, "English"))

S1.4. Google Scholar

Google Scholar does not support controlled vocabulary, truncation, or nested Boolean logic, and limits queries to 256 characters. The following simplified query reflects the documented search terms, applied with a custom date range of 2006 to 2026:

Parkinson "mild cognitive impairment" (tDCS OR "transcranial magnetic stimulation" OR "theta burst" OR "transcranial pulse stimulation" OR "vagus nerve stimulation")

Results were sorted by relevance and screened by title and abstract against the eligibility criteria.

S1.5. Citation tracking

Reference lists of all included studies and of relevant systematic reviews and meta-analyses were screened manually, and forward citation tracking was performed. Citation tracking of a recent meta-analysis of transcutaneous vagus nerve stimulation in Parkinson’s disease (Shan et al., 2025) identified two records, both of which met the inclusion criteria. These records were screened together with the database records.

S1.6. Deduplication

Records were imported into Mendeley, where duplicates were identified automatically and then verified manually in Microsoft Excel by matching title, author, year, and DOI. Of 12,292 records (12,290 from databases and 2 from citation tracking), 8,048 duplicates were removed, leaving 4,244 records for title and abstract screening.
